# UGT2B17 as a predictive biomarker of complete pathological response in HER2 + breast cancer

**DOI:** 10.1007/s12282-026-01840-9

**Published:** 2026-03-06

**Authors:** Ana Gil-Torralvo, M. Ángeles Domínguez-Cejudo, Sonia Molina-Pinelo, Carmen Garrigós, Marta Benavent-Viñuales, Alejandro Falcón, Mónica Cejuela, Beatriz Rodríguez-Alonso, Javier Pascual, Manuel Ruíz-Borrego, Javier Salvador-Bofill

**Affiliations:** 1 Medical Oncology Department, Virgen del Rocio Hospital, Avenida Manuel Siurot sn, Seville, 41013 Spain; 2 Institute of Biomedicine of Seville (IBiS), HUVR, CSIC, Universidad de Sevilla, Seville, Spain; 3 Spanish Center for Biomedical Research Network in Oncology (CIBERONC), Madrid, Spain; 4 Department of Pharmacology, Faculty of Pharmacy, Universidad de Sevilla, Seville, Spain; 5 Medical Oncology Department, Hospital Universitario Reina Sofía, Córdoba/IMIBIC, Córdoba, Spain; 6 Medical Oncology Department, Hospital Universitario Virgen de la Victoria, Málaga, Spain

**Keywords:** HER2-positive breast cancer, Neoadjuvant therapy, Pathological complete response, UGT2B17, Predictive biomarker

## Abstract

**Background:**

Pathological complete response (pCR) after neoadjuvant therapy is a robust surrogate marker for long-term outcomes in breast cancer. Despite major advances with targeted therapies, a significant proportion of patients fail to achieve pCR, underscoring the urgent need for reliable biomarkers that can predict treatment response and guide patient stratification.

**Methods:**

We conducted a two-phase study including 53 patients with HER2-positive, hormone receptor–negative breast cancer treated with neoadjuvant chemotherapy plus anti-HER2 agents. Transcriptomic profiling (discovery cohort, *n* = 13) identified differentially expressed genes associated with therapeutic response, which were validated by qPCR in an independent cohort (*n* = 40). Functional enrichment analysis was performed to explore the biological pathways underlying the differential response.

**Results:**

Differential expression analysis revealed 6251 genes associated with response, with significant enrichment in xenobiotic metabolism and steroid hormone biosynthesis pathways. Within these, members of the UGT2B family (UGT2B10, UGT2B17, UGT2B28) were overexpressed in non-responders. Validation confirmed UGT2B17 (*p* = 0.023, AUC = 0.699, sensitivity 77.2%, specificity 64.5%) and UGT2B28 (*p* = 0.046, AUC = 0.677) as predictive biomarkers of resistance to neoadjuvant therapy. UGT2B17 showing the highest discriminative value.

**Conclusions:**

UGT2B17 overexpression is associated with resistance to neoadjuvant therapy in HER2-positive breast cancer, supporting its potential role as a predictive biomarker. Integration of UGT2B17 into molecular panels could improve patient stratification and guide personalized therapeutic strategies in this subtype.

## Introduction

Pathological complete response (pCR) after neoadjuvant therapy has been established as a robust surrogate marker to predict disease-free survival and overall survival in patients with breast cancer. Patients who achieve pCR have a significant reduction in the risk of recurrence and mortality, with an average 5-year relapse rate of approximately 8% compared with 23% for those patients who do not achieve this response. This finding has established pCR as a key therapeutic endpoint, especially in high-risk subtypes such as HER2-positive breast cancer [1, 2].

Multiple clinical trials shown that the incorporation of targeted therapies, such as trastuzumab and pertuzumab, into chemotherapy regimens containing taxanes and anthracyclines has achieved pCR rates higher than 50% in HER2-positive breast cancer patients, establishing these therapies as the standard neoadjuvant treatment for these patients [1–3]. However, a significant proportion of patients fail to achieve pCR, which reflects the biological heterogeneity of tumors and the need to identify new biomarkers that predict the response to neoadjuvant treatment and improve patient stratification [4–6]. Advances in molecular biology have made it possible to identify genetic alterations associated with the therapeutic response in patients with breast cancer, providing new opportunities for personalized medicine [7, 8]. For example, mutations in *BRCA1* and *BRCA2* have been associated with a higher rate of pCR after neoadjuvant chemotherapy, particularly when platinum-based agents are used [9]. Similarly, HER2 amplification and *TOP2A* alterations have been proposed as predictors of the response to anthracyclines. However, to date, no single validated biomarker that reliably predicts pCR in HER2-positive breast cancer has been identified [10]. Tumor heterogeneity and the complexity of resistance mechanisms continue to be of interest for the optimization of personalized therapeutic strategies in this subtype [11, 12].

In addition to intrinsic factors of the tumor, drug metabolism pathways play important roles in the response to neoadjuvant therapy, since they influence the bioavailability, activation and elimination of therapeutic agents. Conjugation reactions catalyzed by UDP-glucuronosyltransferases (UGTs) represent one of the main pathways involved in the modification and elimination of compounds. The expression and activity of these enzymes are finely regulated in both normal and tumor tissues. Various regulators, including epigenetic factors, endogenous metabolites, and external factors such as diet, the environment, and drugs, affect their expression levels [11, 13]. Variations in these modulations can alter systemic exposure to drugs by modifying the expression of UGT in the tissues responsible for their metabolism, as well as affecting local exposure within tumor tissues. In the context of cancer treatment, acquired resistance to certain drugs may be associated with an induced increase in the expression of UGT, both in preclinical models and in patients with hematological cancers [13].

Different studies suggest that UGT enzymes not only participate in the deactivation of drugs but also directly influence the progression of cancer by modulating endogenous molecules that affect oncogenic pathways, mainly through glucuronidation. Compared with that of normal tissues, tumor status drastically alters the intratumoral expression of UGTs. In some types of cancer, such as prostate, pancreas, lung, endometrial and chronic lymphocytic leukemia (CLL), UGT expression is significantly increased, whereas in other tumors, it is suppressed [14–16]. This variability suggests differential regulation according to the type of cancer.

In breast cancer, particularly in hormone receptor-positive tumors, the enzymes UGT2B15 and UGT2B17 are regulated by the joint action of estrogen (ERα) and androgen (AR) receptors, together with the transcription factor FOXA1 [16]. This hormonal regulation promotes the glucuronidation of sex steroids, modulating key signals in tumor progression. Their high expression has been linked to a better prognosis in certain subtypes of breast cancer, which highlights the role of UGTs as metabolic modulators in this disease [16].

Given the limited understanding of the role of these enzymes in the context of HER2-positive breast cancer, we explored the hypothesis that the expression of genes of the UGT family could be related to the therapeutic response in this subtype. In the present study, we performed a comprehensive gene expression analysis to identify predictive biomarkers of pCR in patients with HER2-positive breast cancer. Our findings highlight the roles of different members of the UGT family, suggesting their possible involvement in therapeutic resistance and their potential utility in optimizing personalized treatment strategies for this subtype of cancer.

## Materials and methods

### Patients

Fifty-three patients with HER2-positive breast cancer and negative hormone receptor expression in localized or locally advanced stages, according to the 8th edition of the classification of the American Joint Committee on Cancer, who were candidates for receiving neoadjuvant treatment, were included. The patients were distributed into two independent cohorts: a discovery cohort (cohort 1) (*N* = 13), which was used for the identification of biomarkers through gene expression analysis, and a validation cohort (cohort 2) (*N* = 40), which was established to confirm the findings obtained in the exploratory phase.

**Fig. 1:**
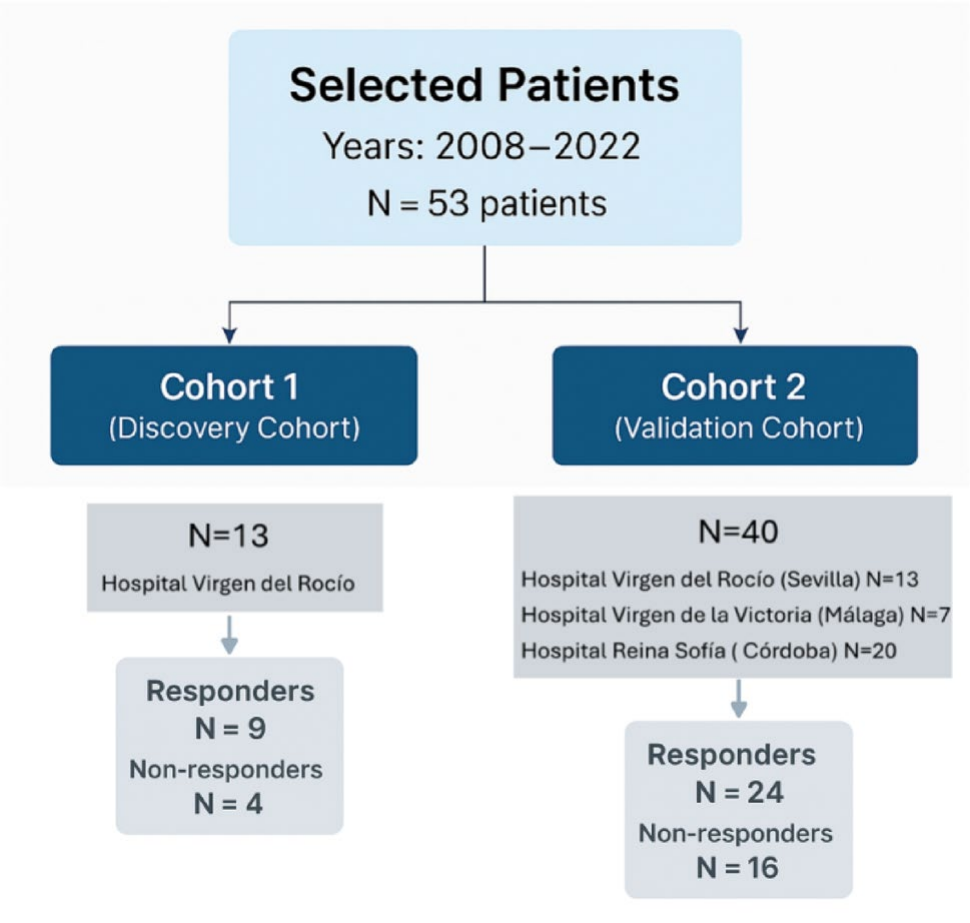
Flowchart of the number of patients included in the study and the hospitals and times at which the samples were collected from the cohorts included in the study

Patients were retrospectively selected between April 2008 and June 2022. Cohort 1 included 13 patients treated at Virgen del Rocío Hospital (Seville, Spain) between 2008 and 2017. Cohort 2 included 40 patients treated between 2011 and 2022 in three Spanish hospitals: Virgen del Rocío Hospital (Seville, *N* = 13), Virgen de la Victoria Hospital (Málaga, *N* = 7) and Reina Sofía Hospital (Córdoba, *N* = 20). The inclusion of patients in our study is detailed in the flow diagram presented in Fig. 1.

The neoadjuvant treatment they received consisted of standard chemotherapy (anthracyclines and/or taxanes) combined with anti-HER2 agents alone (trastuzumab) or in combination (trastuzumab and pertuzumab). The patients were stratified according to their response to neoadjuvant treatment according to the residual cancer burden (RCB) classification. The responders were those who achieved a complete pathological response (RCB-0), whereas the nonresponders included patients with residual disease after treatment (RCB-I, II or III).

Detailed clinical and pathological data, including age at diagnosis, clinical tumor stage, body mass index (BMI), percentage of patients with Ki67 expression, treatment received, histopathological response, and severe therapy-related toxicity, were collected. This characterization allowed an exhaustive analysis of the clinical and biological factors associated with the response to treatment.

## RNA extraction

RNA extraction was performed from 8 to 10 μm sections of formalin-fixed paraffin-embedded (FFPE) samples obtained from diagnostic biopsies using a RecoverAll™ Total Nucleic Acid Isolation Kit (Thermo Fisher Scientific, Waltham, MA, USA) according to the manufacturer’s recommendations with modifications in the protease digestion stage, where prolonged incubation at 50 °C for three hours was used to optimize the recovery of nucleic acids. The RNA samples obtained were stored at -80 °C until use. The concentrations of the RNA samples were quantified using a NanoDrop spectrophotometer (Thermo Fisher Scientific, Waltham, MA, USA).

## Gene expression analysis and selection of differentially expressed genes

Gene expression analysis in Cohort 1 was carried out using a Clariom D microarray (Thermo Fisher Scientific, Waltham, MA, USA), which is based on Affymetrix microarray technology. Washing and scanning were performed using the Affymetrix GeneChip system, which includes a GeneChip 645 Hybridization Oven, a GeneChip 450 Fluid Station and a GeneChip 7G Scanner.

Gene expression data were normalized and analyzed using the Transcriptome Analysis Console (TAC) 4.0 software (Thermo Fisher Scientific). To identify differentially expressed genes between patients with and without pCR, the criterion of a change in expression greater than 1.5 or less than − 1.5 in terms of fold change was applied, with a p value less than 0.05.

### Data availability

The microarray transcriptomic data generated in the discovery cohort have been deposited in the NCBI Gene Expression Omnibus (GEO) under accession number GSE310900. Reviewer access to the dataset is available during peer review through the GEO private token provided to the journal.

### Functional enrichment analysis

Functional enrichment analysis was performed on the differentially expressed genes. For this purpose, the DAVID tool (Database for Annotation, Visualization, and Integrated Discovery) (david.ncifcrf.gov) was used. The analysis was subsequently carried out using the Kyoto Encyclopedia of Genes and Genomes (KEGG) database, which integrates genomic, chemical and biological system information. Pathways with a false discovery rate (FDR ) < 0.05 and a fold change > 2 were considered significantly enriched. The visualization of the functional enrichment results was performed using R (version 4.2.2, R Core Team, Vienna, Austria) and the complementary packages ggplot2, ggsankey and ggalluvial, which made it possible to generate Sankey-type flow diagrams and bubble graphs of significantly enriched pathways [17].

### Validation using quantitative real-time PCR (qPCR)

Validation of the selected genes in cohort 1 was performed in cohort 2. For this purpose, 10 µL of total RNA was reverse-transcribed to complementary DNA (cDNA) using the High Capacity cDNA Reverse Transcription Kit (Applied Biosystems, Foster City, CA, USA). The cDNA was subsequently preamplified with TaqMan^®^ PreAmp Master Mix (Thermo Fisher Scientific, Waltham, MA, USA), and gene expression was quantified by real-time PCR (qPCR) using specific TaqMan^®^ gene expression assays: B2M (Hs00984230_m1), UGT2B10 (Hs02556263_s1), UGT2B17 (Hs04194440_g1) and UGT2B28 (Hs03044951_m1) (Applied Biosystems). Amplification reactions were performed in a final volume of 20 µL, including 10 µL of TaqMan^®^ Universal PCR Master Mix, 1 µL of the corresponding TaqMan assay, and 9 µL of diluted preamplified cDNA. The cycling conditions were as follows: initial denaturation at 95 °C for 10 min, followed by 40 cycles of 95 °C for 15 s and 60 °C for 1 min, and each sample was analyzed in triplicate. The B2M gene was used as an endogenous control to normalize the relative expression of the genes of interest, applying the 2 ^ −ΔCt method, which was calculated as follows: ΔCt = Ct (gene of interest) - Ct (B2M) [18].

### Statistical analysis

Descriptive statistics were used to characterize the most relevant clinical parameters. The normality of the continuous variables was evaluated using the Shapiro‒Wilk test. For those that followed a normal distribution, Student’s t test was applied for independent samples, while the variables that did not meet this criterion were analyzed with the Mann‒Whitney U test. The categorical variables were compared using the chi-square test.

To evaluate the potential of the selected genes as predictive biomarkers of pCR, an analysis of receiver operating characteristic (ROC) curves was performed to determine the optimal cutoff point of expression that maximized sensitivity and specificity, allowing effective differentiation between responders and nonresponders to treatment.

Statistically significant results were those with a p value lower than 0.05. All the statistical analyses were carried out using SPSS software, version 28 (IBM Corp., Armonk, NY, USA).

## Results

### Characteristics of the study population

The clinical and tumor characteristics and the treatments administered in both cohorts are detailed in Table 1. No significant differences were observed between the cohorts in terms of age at diagnosis, clinical tumor stage (TNM), percentage of patients with Ki67 expression, type of treatment received, associated severe toxicity or pCR rate. The mean age at diagnosis was 63 years [interquartile range (IQR): 33–70] in cohort 1 and 56 years [IQR range: 38–81] in cohort 2 (*p* = 0.432). Most of the patients in both cohorts had an advanced tumor stage (IIB–IIIC), with no statistically significant differences (*p* = 0.145).

**Table 1:** Comparison of clinical and tumor characteristics, treatment, toxicity and therapeutic response between cohorts 1 and 2

Variables	Cohort 1, *n* (%) (*N* = 13)	Cohort 2, *n* (%) (*N* = 40)	*P* value
Age, median years (IQR)	63 (33–70)	56 (38–81)	0.432
ECOG home			0.411
ECOG 0	13 (100)	38 (95)	
ECOG 1	0	2 (5)	
Menopausal state			0.234
Premenopausal	2 (15.4)	13 (32.5)	
Menopausal	11 (84.6)	27 (67.5)	
Obesity (IMC ≥ 30)			0.020
IMC ≥ 30	4 (30.8)	27 (67.5)	
IMC < 30	9 (69.2)	13 (32.5)	
Clinical TNM			0.145
AI	0	1 (1.9)	
IIA	2 (15.4)	7 (17.5)	
IIB	3 (23.1)	13 (32.5)	
IIIA	8 (61.5)	11 (27.5)	
IIIB	0	7 (17.5)	
IIIC	0	1 (2.5)	
Ki67			0.868
≥ 50%	8 (61.5)	25 (64.1)	
< 50%	5 (38.5)	15 (37.5)	
Treatment received			NA
Anthracyclines, taxanes, trastuzumab	8 (61.5)	8 (20)	
Trastuzumab, taxanes	5 (38.5)	0	
Anthracyclines, taxanes, trastuzumab, pertuzumab	0	29 (72.5)	
Pertuzumab, trastuzumab, taxanes	0	3 (7.5)	
Toxicity 3 or 4			0.398
Yes	2 (15.4)	3 (7.5)	
No	11 (84.6)	37 (92.5)	
Complete pathological response (PCR)			0.551
Yes	9 (69.2)	24 (60)	
No	4 (30.8)	16 (40)	
Degree of response (RCB)			0.426
RCp	9 (69.2)	24 (60)	
RCB I	0	7 (17.5)	
RCB II	3 (23.1)	6 (15)	
RCB III	1 (7.7)	3 (7.5)	

*BMI* body mass index, *ECOG* eastern cooperative oncology group performance status, *RCB* residual cancer burden

However, significant differences were identified in the prevalence of obesity ( BMI ≥ 30), which was greater in cohort 2 (67.5%) than in cohort 1 (30.8%) (*p* = 0.020). In addition, the differences in the therapeutic regimens were notable, since a considerably greater percentage of patients in cohort 2 received pertuzumab in combination with anthracyclines, taxanes and trastuzumab (72.5%) than did those in cohort 1, where this regimen was not administered due to the later approval of pertuzumab.

Regarding the response to treatment, the pCR rate was 69.2% in cohort 1 and 60% in cohort 2, with no significant differences between the two groups (*p* = 0.551). Similarly, the distribution of the degree of response according to the residual cancer burden (RCB) classification showed that the majority of patients in both cohorts achieved a pCR or a low RCB (RCB I-II), without significant differences (*p* = 0.426).

### Differential gene expression analysis in the discovery cohort

To identify potential biomarkers associated with the response to neoadjuvant treatment, a transcriptomic analysis was performed in the discovery cohort using a Clariom D microarray from Affymetrix. This technology allows the evaluation of more than 20,000 human transcripts, including coding and noncoding genes, as well as alternative splicing events, providing a detailed expression profile.

All microarray data from the discovery cohort are publicly archived in the GEO repository (accession GSE310900).

**Fig. 2:**
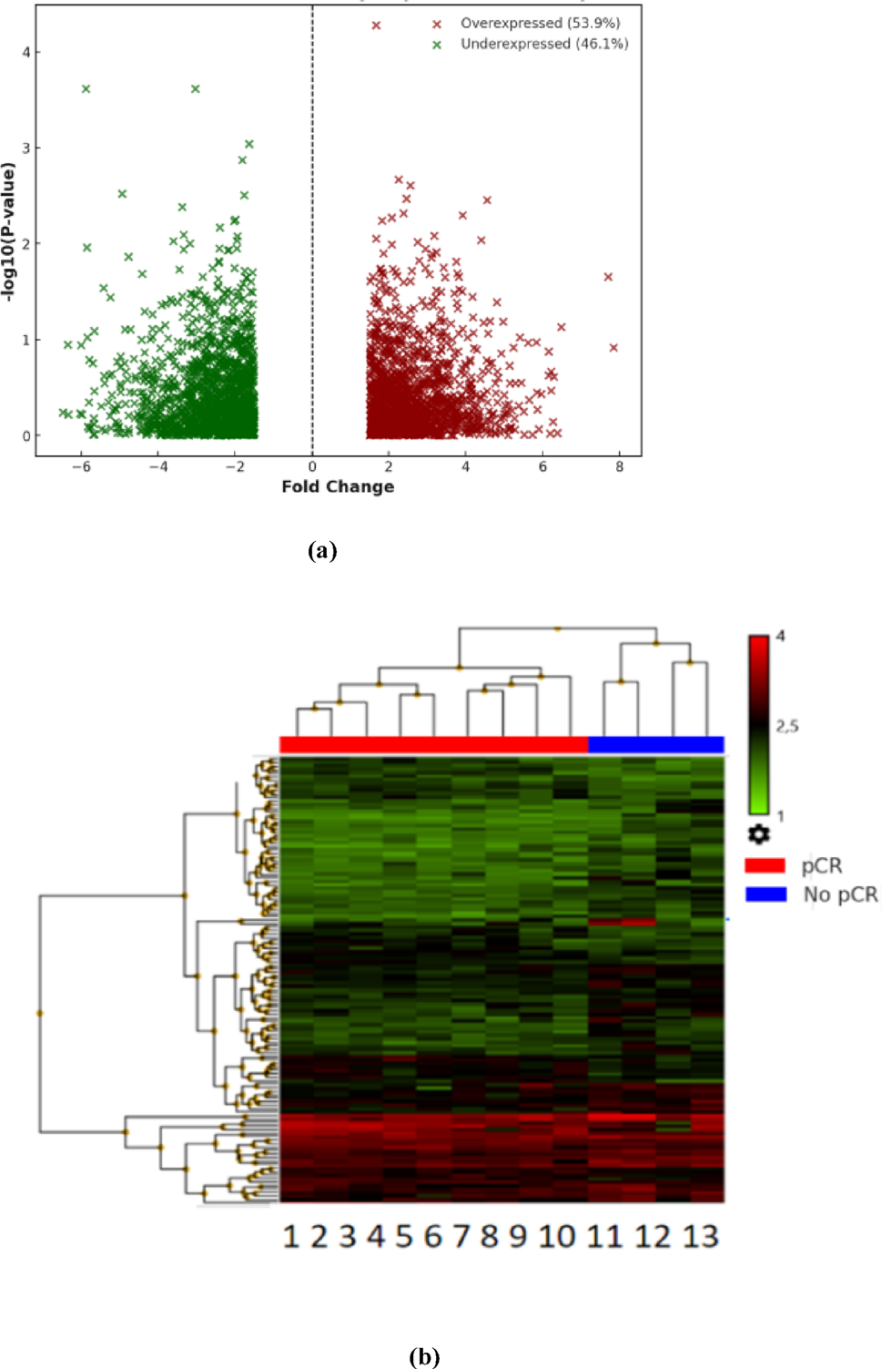
**a** Differential gene expression analysis between patients with and without CRp. **a** Volcano plot: The X axis represents the log₂ (fold change) of gene expression, whereas the Y axis represents the –log₁₀ (p value), reflecting the statistical significance of each transcript. Upregulated genes in CRp patients are shown in red on the right side of the graph, whereas the downregulated genes in this group are shown in green on the left side. **b** Two-dimensional heatmap of differentially expressed genes between patients with a complete pathological response (RCP, red) and patients without a complete pathological response (noRCP, blue) to neoadjuvant treatment. Each column represents an individual patient sample, and each row represents a gene. The color scale indicates the relative expression levels: red corresponds to upregulated genes, whereas green indicates downregulated genes compared with the average expression of the dataset. The upper dendrogram shows the hierarchical grouping of the samples according to their transcriptomic profile, revealing a clear separation between the RCP and noRCP groups

The results of the transcriptomic analysis are presented in Fig. 2. Using an ANOVA model (*p* < 0.05, fold change ≥ 1.5), a total of 6251 differentially expressed genes were identified between patients who were responsive and nonresponsive to neoadjuvant treatment. Among these genes, 3367 (53.9%) were overexpressed and 2884 (46.1%) were underexpressed in nonresponders compared with responders (Fig. 2a). Additionally, the unsupervised hierarchical grouping analysis generated a dendrogram in the form of a heatmap, where the samples were grouped by the studied conditions (pCR and No pCR), suggesting different gene expression profiles between the populations (Fig. 2b).

**Fig. 3:**
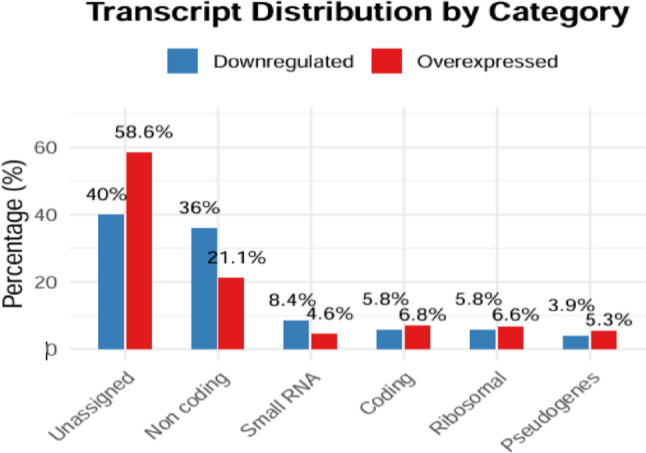
Functional classification of differentially expressed genes

Subsequently, a functional classification of the differentially expressed genes was carried out between patients with and without pCR (Fig. 3). Among the 386 identified protein-coding genes, 222 were upregulated and 164 were downregulated in nonresponders compared with those who achieved pCR. On the other hand, 389 ribosomal genes were identified, which represented approximately 22% of the total analyzed genes. These genes are associated with the protein synthesis machinery and its regulation. Three hundred ninety-seven genes were classified as small RNAs, including microRNAs (miRNAs) and other small noncoding RNAs, which play crucial roles in the posttranscriptional regulation of gene expression, were also identified. In addition, 290 pseudogenes were found, which represented approximately 4.6% of the total genes analyzed. Finally, a group consisting of 3127 genes (approx. 50%) could not be classified into any of the previous categories, which suggests the presence of long noncoding RNAs (lncRNAs) or other RNA species.

### Functional enrichment analysis of protein-coding differentially expressed genes between responders and nonresponders to neoadjuvant therapy

**Fig. 4:**
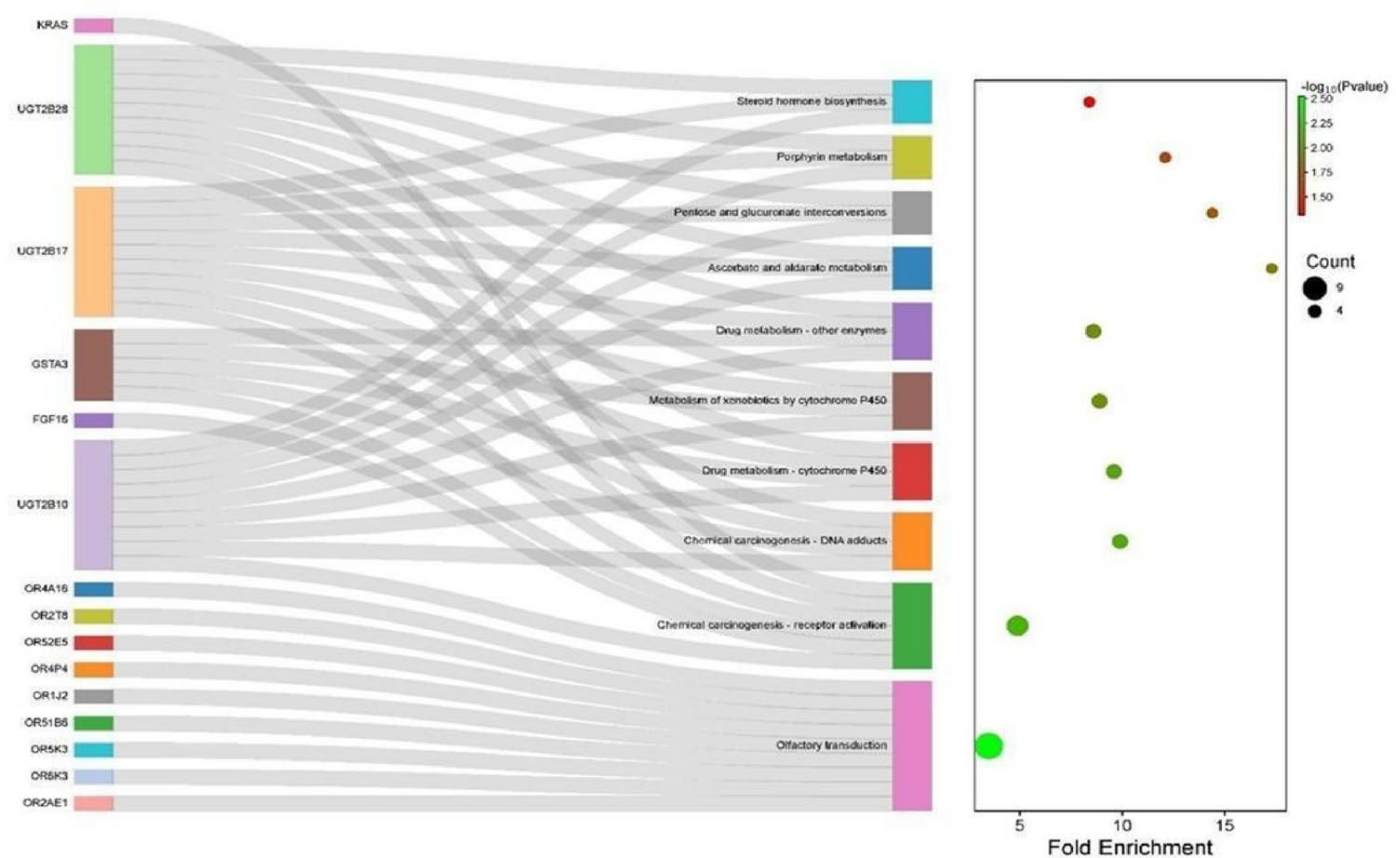
Functional enrichment analysis of the differentially expressed genes in cohort 1. On the left is a Sankey diagram that visualizes the relationship between the identified genes and the significantly enriched metabolic pathways, whereas on the right is a bubble chart indicating the degree of enrichment (fold change) and the adjusted statistical significance (–log10 (p value))

A functional enrichment analysis of the 386 identified protein-coding genes was performed to explore the enriched biological pathways. The results revealed that a subset of these genes were associated with 10 metabolic pathways (Fig. 4), including ascorbic acid metabolism and aldarate, pentose and gluconate conversion, porphyrin metabolism, chemical carcinogenesis, cytochrome P450 drug and xenobiotic metabolism, drug metabolism mediated by other enzymes, steroid hormone biosynthesis, and olfactory transduction.

Ten metabolic pathways that met the predetermined significance criterion (adjusted FDR) were revealed to be enriched in several genes, whereas no relevant association was observed (“no output”) for 113 genes, revealing that the majority do not show a direct link with the pathways analyzed under the established thresholds.

Notably, the UGT2B family (specifically UGT2B10, UGT2B17 and UGT2B28) was identified as a group of special interest in this analysis since the results of the functional enrichment analysis revealed that these genes participate in critical pathways, such as xenobiotic metabolism and steroid hormone biosynthesis. These findings led to specific analyses of these genes.

### Differential expression of the UGT2B10, UGT2B17 and UGT2B28 genes between responders and nonresponders in the discovery cohort

Once the subgroup of genes of the UGT2B family were found to be of interest in the functional enrichment analysis, we proceeded to evaluate the differences in their expression between responders and nonresponders in the discovery cohort.

**Fig. 5:**
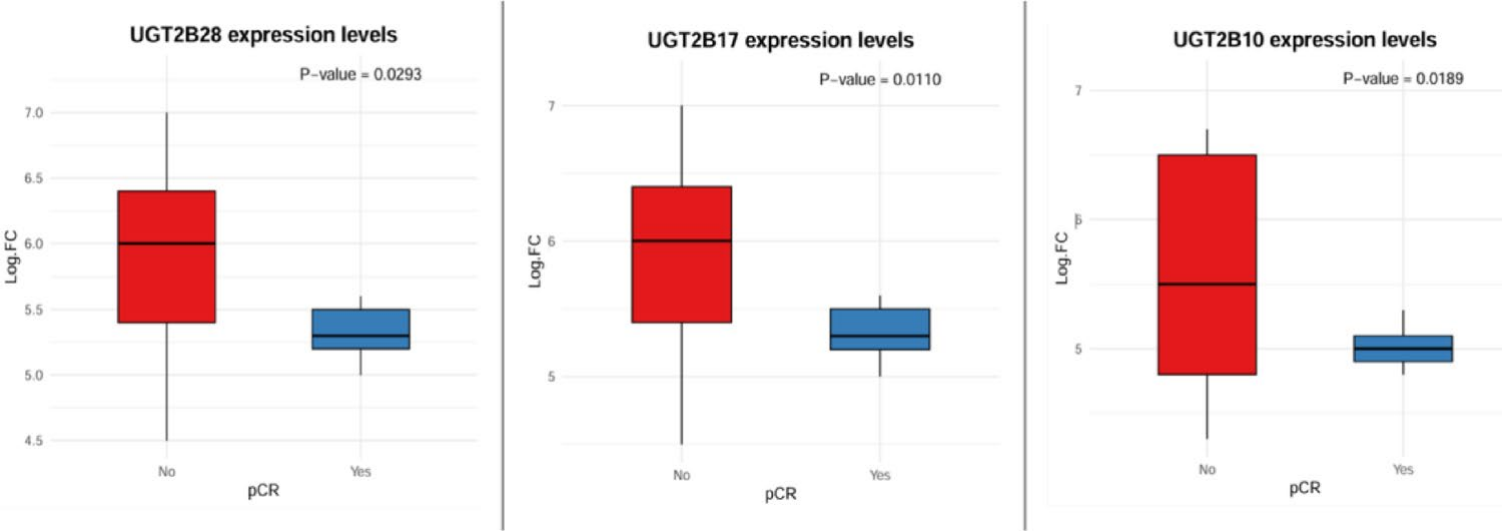
Relationships between the expression of UGT2B28, UGT2B17 and UGT2B10 and complete response in the discovery cohort

A microarray analysis revealed that UGT2B10, UGT2B17 and UGT2B28 were significantly upregulated in nonresponders compared with responders, supporting their involvement as potential biomarkers of resistance (Fig. 5).

### Validation of the expression of UGT2B10, UGT2B17 and UGT2B28 and their ability to predict pCR

**Fig. 6:**
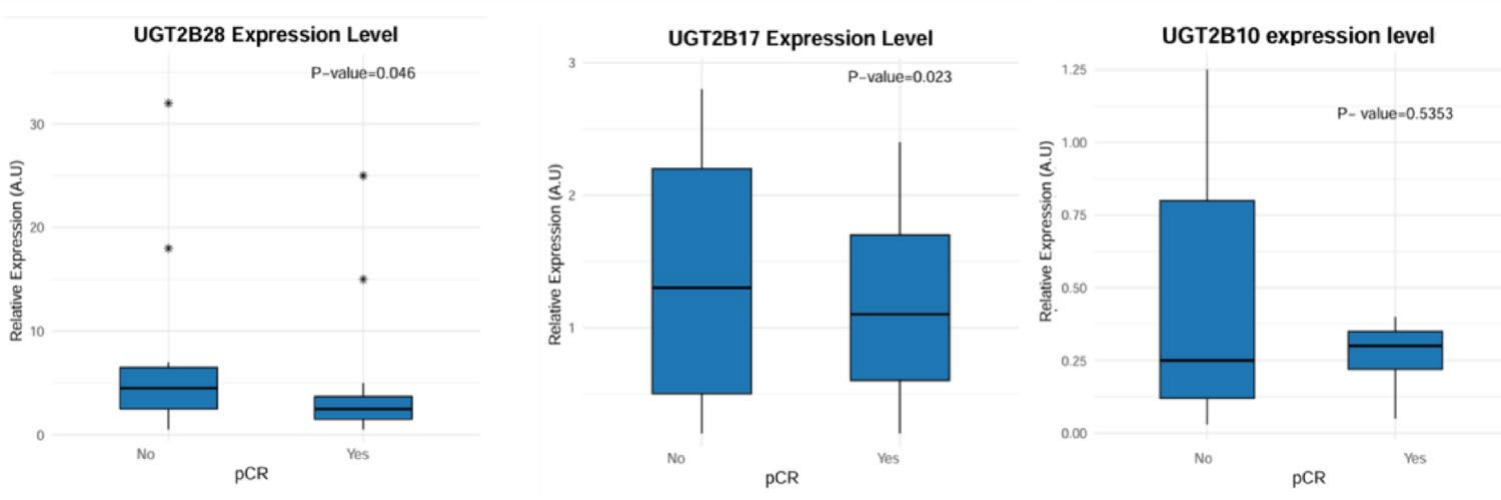
Relative expression of UGT2B28, UGT2B17 and UGT2B10

qPCR analysis of these genes in the validation cohort revealed statistically significant differences in their expression levels between responding and nonresponding patients only for the UGT2B17 genes, with a p value of 0.023, and B28, with a p value of 0.046, confirming the relevance of the initial findings in cohort 1, whereas no significant differences were found for the UGT2B10 gene (p value of 0.535). The results of the qPCR performed in the validation cohort are shown in Fig. 6.

**Fig. 7:**
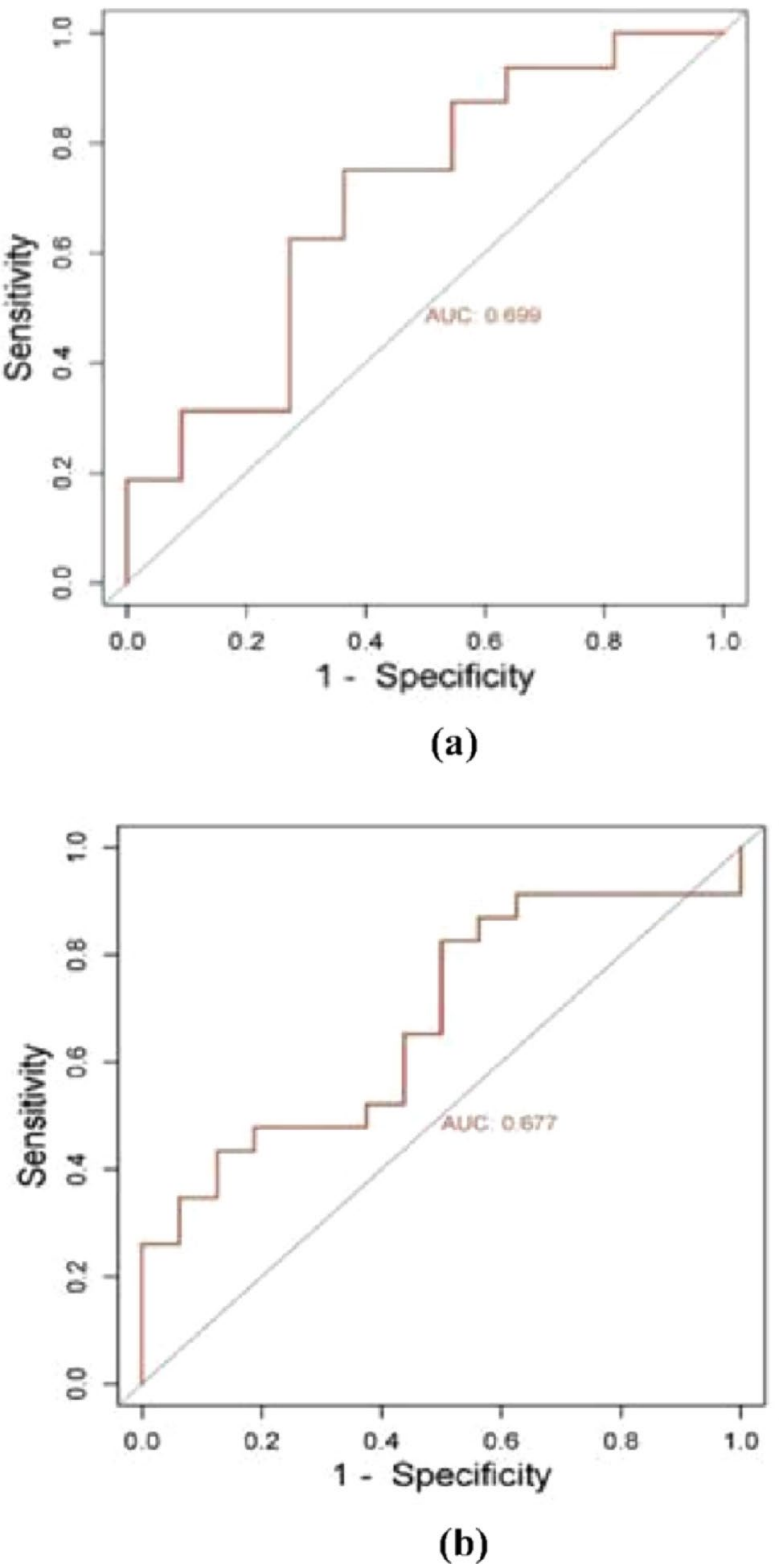
ROC curves for the evaluation of the predictive performance of UGT2B17 and UGT2B28 as biomarkers of therapeutic response. **a** ROC curve of UGT2B17. **b** ROC curves of UGT2B28

ROC curve analysis was carried out only for the significant genes in the validation cohort, UGT2B17 and UGT2B28, to evaluate their predictive value of CRp, determining its sensitivity and specificity as a potential biomarker (Fig. 7). Among both genes, UGT2B17 presented a superior discriminative capacity, with an AUC value of 0.699. The sensitivity and specificity were 77.2% and 64.5%, respectively, suggesting that the overexpression of this gene could be associated with a lower probability of reaching a CRp. These results were statistically significant, with a p value = 0.023 and a 95% confidence interval (0.516–0.928) (Fig. 7a). On the other hand, although UGT2B28 also showed differences in expression between the groups studied (*p* = 0.046), its discriminative capacity was lower than that of UGT2B17, with an AUC value = 0.677 (Fig. 7b).

## Discussion

The present study provides a comprehensive view of the role of the UGT2B family, particularly UGT2B17, as a possible biomarker of resistance to neoadjuvant treatment in HER2-positive and HR-negative breast cancer. Through a sequential approach based on the analysis of gene expression using a microarray in the discovery phase, more than 6,200 differentially expressed genes were identified between patients with and without a response to treatment, which highlights the great molecular heterogeneity of HER2-positive breast cancer [19, 20]. The identification of altered metabolic pathways in treatment-resistant tumors has significant biological and clinical relevance since these molecular circuits represent functional nodes where the processes of detoxification, drug biotransformation and tumor metabolic reprogramming converge. Alterations in pathways such as xenobiotic metabolism and steroid hormone biosynthesis not only affect the intratumoral pharmacokinetics of chemotherapeutic agents but also modulate the cellular microenvironment, affecting proliferation, apoptosis and the ability to evade therapies. The overexpression of genes in the UGT2B family in this context could not only promote the inactivation of cytotoxic drugs by glucuronidation but also modulate the levels of steroids and bioactive metabolites, favoring resistance phenotypes [13, 21, 22]. Our analysis confirmed the existence of clearly differentiated gene expression profiles according to the therapeutic response, which reinforces the hypothesis that resistance to neoadjuvant therapy is determined by complex and multifactorial molecular mechanisms. Among the protein-coding genes, the UGT2B family (especially UGT2B17, UGT2B28 and UGT2B10) was upregulated in nonresponders, with UGT2B17 showing the highest discriminative value in the validation cohort (AUC 0.699, sensitivity 77.2%, specificity 64.5%) [19, 20].

UGT enzymes can contribute to drug resistance in cancer through glucuronidation, a biotransformation process that inactivates lipophilic compounds, including cytotoxic drugs, facilitating their elimination from the body. This mechanism is widely related to drug resistance in various types of cancer, both in the context of intrinsic and acquired resistance, including cytostatics and targeted therapies [13, 23–25]. The activity of UGTs converts drugs into more polar metabolites, which are better substrates for efflux transporters, contributing to multidrug resistance [24, 25].

UGT2B17 was significantly upregulated in patients who did not respond to neoadjuvant treatment compared with those who did, suggesting its involvement in the modulation of the therapeutic response. UGT2B17 is involved in the metabolism of both drugs and steroid hormones. UGT2B17 shows high interindividual variability in its expression and activity, as determined by genetic polymorphisms and variations in the number of copies, and is responsible for the glucuronidation of testosterone, dihydrotestosterone and numerous drugs, especially in the liver and intestine. The activity of UGT2B17 is correlated with its ability to metabolize androgens and drugs, which may influence the therapeutic response and pharmacokinetics of antineoplastic agents [26–28]. Specifically, UGT2B17 participates in the glucuronidation of drugs such as anthracyclines, which are used in standard neoadjuvant regimens. Upregulation of UGT2B17 could reduce the intracellular bioavailability of drugs and, therefore, compromise their cytotoxic effectiveness [29].

Likewise, tumor metabolic reprogramming could play a relevant role, since the overexpression of UGT2B17 can contribute to the inactivation of endogenous metabolites such as steroids, and bioactive lipids, altering the tumor microenvironment and activating signaling pathways associated with cell proliferation, survival and immune evasion. Notably, in our functional enrichment analysis, UGT2B17 and UGT2B28 participate in critical metabolic pathways such as xenobiotic metabolism and steroid hormone biosynthesis, processes previously associated with mechanisms of therapeutic resistance and tumor metabolic reprogramming [13, 32].

From a biological standpoint, UGT2B17 overexpression could contribute to reduced efficacy of anthracycline-based regimens by increasing glucuronidation of doxorubicin and related metabolites, thereby lowering intracellular exposure and facilitating efflux of more polar conjugates. In parallel, UGT2B17 may influence HER2-positive tumor biology by reshaping intratumoral steroid availability; glucuronidation of androgens could dampen AR signaling in AR-high tumors or, conversely, reflect an adaptive AR/FOXA1-driven transcriptional program previously described in breast cancer [30, 31].

With respect to UGT2B28 as a possible modulator, although it did not show the most powerful discrimination in our cohort, its overexpression in nonresponding patients and its demonstrated participation in the metabolism of steroids and xenobiotics suggest a possible complementary or modulatory role in the mechanisms of therapeutic resistance. UGT2B28 catalyzes the glucuronidation of steroid hormones such as estradiol, estrone, androsterone and other lipophilic compounds, facilitating their elimination and altering their intracellular levels [33]. Its expression has also been detected in breast tissue, and its regulation seems to be mediated by hormonal signals such as androgen receptors and growth factors (EGF, GR) [34].

Although UGT2B17 appears to be the predominant marker, the concomitant presence of UGT2B28 strengthens the notion that a UGT2B family is involved in drug resistance, increasing the robustness of these findings.

Although the findings of the present study offer a solid basis that suggests the involvement of UGT2B17 in resistance to neoadjuvant treatment, further in vitro and in vivo functional studies that directly evaluate the impact of UGT2B17 upregulation on the sensitivity to specific drugs are needed.

Notably, resistance to treatment is a multifactorial phenomenon. In addition to metabolic mechanisms mediated by UGTs, factors such as tumor heterogeneity, interactions with the microenvironment, and the presence of specific immunological signatures can also modulate the response therapy. In this context, the integration of UGT2B17 within multigene biomarker panels, which include molecular and immunological factors, could optimize patient stratification and favor a more personalized therapeutic approach.

Among the limitations of our study are the small size of the discovery cohort. The discovery cohort was necessarily limited because, after the initial samples had been processed, pertuzumab became incorporated into routine neoadjuvant regimens, and we prioritized an independent validation cohort treated in the contemporary setting. Notably, similar translational biomarker studies frequently include modest sample sizes due to tissue availability and assay requirements [35]. In addition, variability in the therapeutic regimens administered—especially the introduction of pertuzumab in the validation cohort—and the high prevalence of obesity in the validation cohort could have influenced both the observed gene expression and the response to treatment. Furthermore, the absence of functional studies limits the possibility of establishing a direct causal relationship between the upregulation of UGT2B17 and UGT2B28 and resistance to treatment. Although the transcriptomic findings and their validation support a robust association, only functional confirmation will make it possible to rule out indirect effects or epiphenomena. We also acknowledge that androgen receptor status and intrinsic molecular subtyping were not available; given the link between UGT2B17 and steroid/androgen metabolism, these unmeasured factors might also have influenced the observed association.

In conclusion, the overexpression of UGT2B17 in patients who do not respond to neoadjuvant therapy in HER2-positive breast cancer suggests that this gene could function as a biomarker of therapeutic resistance. Our findings underscore the importance of the integration of transcriptomic profiles to advance personalized medicine in HER2-positive breast cancer, allowing the identification of patients with a greater risk of resistance and optimizing therapeutic strategies. Although the upregulation of UGT2B17 is presented as the most consistent finding as a biomarker of resistance, the additional implication of UGT2B28 supports the existence of a UGT2B gene family with a possible modulatory role in drug resistance, which suggests that both genes could be incorporated together in predictive panels.
